# Optimization of Cd (II) removal from aqueous solution by natural hydroxyapatite/bentonite composite using response surface methodology

**DOI:** 10.1038/s41598-023-32413-x

**Published:** 2023-03-29

**Authors:** Yiene Molla Desalegn, Endrias Adane Bekele, Femi Emmanuel Olu

**Affiliations:** 1 Department of Mechanical Engineering, School of Mechanical and Chemical Engineering, Woldia Institute of Technology, Woldia University, Woldia, Ethiopia; 2 Faculty of Materials Science and Engineering, Jimma Institute of Technology, Jimma University, Jimma, Ethiopia

**Keywords:** Engineering, Materials science

## Abstract

Toxic cadmium (Cd) was removed from water using eggshell-based hydroxyapatite (HAp) grafted bentonite (HAp/bentonite) composite through a straightforward chemical synthesis route. The as-prepared adsorbents were characterized using X-ray diffraction (XRD), scanning electron microscopy (SEM), Fourier transform infrared spectroscopy (FTIR), and Brunauer–Emmett–Teller analysis (BET). Optimization of the initial adsorbate concentration, adsorbent dosage, pH, and contact time—all of which affect the adsorption process—was performed using the central composite design (CCD) of the response surface methodology (RSM). 99.3 percent adsorptive removal efficiency was observed at an initial concentration of 61.58 mg/L of Cd (II), with an adsorbent dosage of 1.58 g, a solution pH of 5.88, and a contact time of 49.63 min. The analysis of variance (ANOVA) was performed, and the multiple correlation coefficient (R^2^) was found to be 0.9915 which confirms the significance of the predicted model. The Langmuir isotherm model best represented the adsorption isotherm data, which also predicted a maximum sorption capacity of 125.47 mg/g. The kinetic data were best described by the pseudo-second order model.

## Introduction

Heavy metal ion pollution of water is now the most concerning environmental problem because of its devastating effects on both nature and humans. Lead (Pb), copper (Cu), cadmium (Cd), chromium (Cr), mercury (Hg), arsenic (As), zinc (Zn), and nickel (Ni) are all examples of heavy metals because their densities are higher than 5 g/cm^3^^1–3^. Electroplating, metallurgy and mining, agriculture, dying, coking, pharmaceuticals, and nickel–cadmium batteries are just some of the industries and products that release toxic heavy metals into the environment^4^. Cd(II) is the most important of these^5^. Cd(II) is readily absorbed by ecosystems and then accumulates in people through the food chain, where it has been linked to cancer, hypertension, renal dysfunction, and liver disorders^6^. Potable water should not contain more than 0.003 mg/L of Cd(II), as recommended by the World Health Organization^7,8^. For obvious reasons, heavy metal contaminants in aqueous solutions must be eliminated using appropriate technologies, such as adsorption, coagulation, flocculation, ion exchange, bioremediation, and membrane separation^9^. Adsorption stands out as the best treatment option because of its low cost, high selectivity, high efficiency, and lack of hazardous byproducts^10,11^.

Recently, clays have drawn the attention of environmental researchers due to their availability, cost-effectiveness, and chemical and mechanical stability^12^. For application in heavy metal removal from wastewater, the clay needs to possess a large surface area, chemical stability, and high cation exchange capacity^13,14^. Due to its large specific surface area and relatively high cation-exchange capacity, bentonite was considered an efficient adsorbent in the removal of various heavy metal ions.

Hend et al. (2016) evaluated the application of nano-bentonite to remove Pb(II) and Cd(II) from an aqueous solution. The results revealed that the adsorption behavior of Pb(II) and Cd(II) onto nano-bentonite was best described with the Langmuir isotherm model, with a maximum adsorption capacity of 67 and 83 mg/g, respectively^15^.

Şahan (2019) used bentonite enriched with SH groups (BSH) to remove Pb(II) and Cd(II) ions. In this study, the effect of different input variables (pH, initial metals concentration, adsorbent dosage, and contact time) on the removal efficiency was estimated. The optimal condition for pH, initial concentration, adsorbent dosage, and contact time was 5.10, 32.98 mg/L, 146 mg, and 146.01 min. for Pb(II), while these values were 4.52, 37.9 mg/L, 152.3 mg, and 146.12 min for Cd(II), respectively. At the optimum values, maximum Pb(II) and Cd(II) removal were obtained as 95.05% and 91.4%, respectively^16^.

In another study, Zhou et al. (2019) developed amino-functionalized bentonite/CoFe_2_O_4_@MnO_2_ for the adsorption of Cd(II). The results revealed that the maximal adsorption efficiency for Cd(II) was found to be 98.88%. Moreover, The equilibrium adsorption isotherm showed that Langmuir isotherm fitted well to the experimental data with a maximum adsorption capacity of 115.79 mg/g^17^. However, the adsorption capacity of bentonite towards heavy metals ion may be limited by a lack of surface functional groups.

Over the past decades, apatite minerals like hydroxyapatite (HAp, Ca_10_(PO_4_)_6_(OH)_2_, has attracted huge interest in effectively adsorbing heavy metals ion^18,19^. Some previous studies investigated HAp for the removal of a variety of contaminants. Nu´nez et al. (2019) studied the adsorptive removal of heavy metals ions using HAp, and the highest adsorption capacities were found to be 265, 64, and 55 mg/g for Pb(II), Cd(II), and Cu(II), respectively^20^. It is worth mentioning that the high ion exchange capacity and the ability to form a dissolution–precipitation reaction leads HAp to be one of the most competitive adsorbents in wastewater treatment^21^. Despite these interesting attributes, the high surface activity of HAp nanoparticles can lead to the formation of aggregates in aqueous solutions due to van der Waal’s forces, which lowers the surface area and hinder the practical application of HAp for removing heavy metals from wastewater^22^. Furthermore, the application of synthetic HAp for wastewater treatment is constrained by its relatively high production cost^23–25^. Therefore, studies are urgently needed on the improvement of HAp with a large surface area for adsorptive removal of Cd(II) from water. In this regard, bentonite clay can improve the surface area and cation-exchange capability, which in turn facilitates the reaction of HAp towards Cd(II)^26^.

Hence, in this study, a new composite from eggshell-based HAp and bentonite was developed by a simple chemical synthesis route using glutaraldehyde as a cross-linking agent. The entire synthesis process was conducted under mild experimental conditions without using specialized equipment while consuming easily available and cost-effective starting reagents. Specifically, compared with the chemical reagents, eggshell had the significant advantage of being the cheepest source of hydroxyapatite. besides, effective re-utilization of waste materials for decontamination of water is also the basic principle of waste recycling.

Additionally, in the traditional method of analyzing the adsorption process, the intractive effect of process parameters on each other is not considered i.e., only one parameter is changed, and the other parameters are kept constant at a non-optimal level, which results in a cost and time-consuming and tedious optimization process. In this connection, the response surface methodology (RSM) was utilized, which is based on a set of statistical and mathematical techniques to design the experimental runs. Moreover, statistical optimization of operating parameters such as the initial adsorbate concentration, adsorbent dosage, pH, and contact time was conducted using a central composite design (CCD) of response surface methodology (RSM). The adsorption kinetics and isotherm modeling were also investigated to analyze the adsorption mechanism.

## Experimental

### Chemicals and reagents

All chemicals and reagents such as the phosphoric acid (H_3_PO_4_, 85%), ammonium hydroxide (NH_4_OH), sodium hydroxide (NaOH, 99.8), glutaraldehyde (C_5_H_8_O_2_, 25%), and cadmium chloride (CdCl_2_, 99%) were used in this experiment without any further purification. Bentonite powder was purchased from Alkane Chemicals P.L.C (Addis Ababa, Ethiopia) and the eggshell was collected from the students’ cafeteria of Jimma University (Jimma, Ethiopia). All the solutions were prepared using Distilled water.

### Hydroxyapatite (HAp) preparation

**Figure 1:**
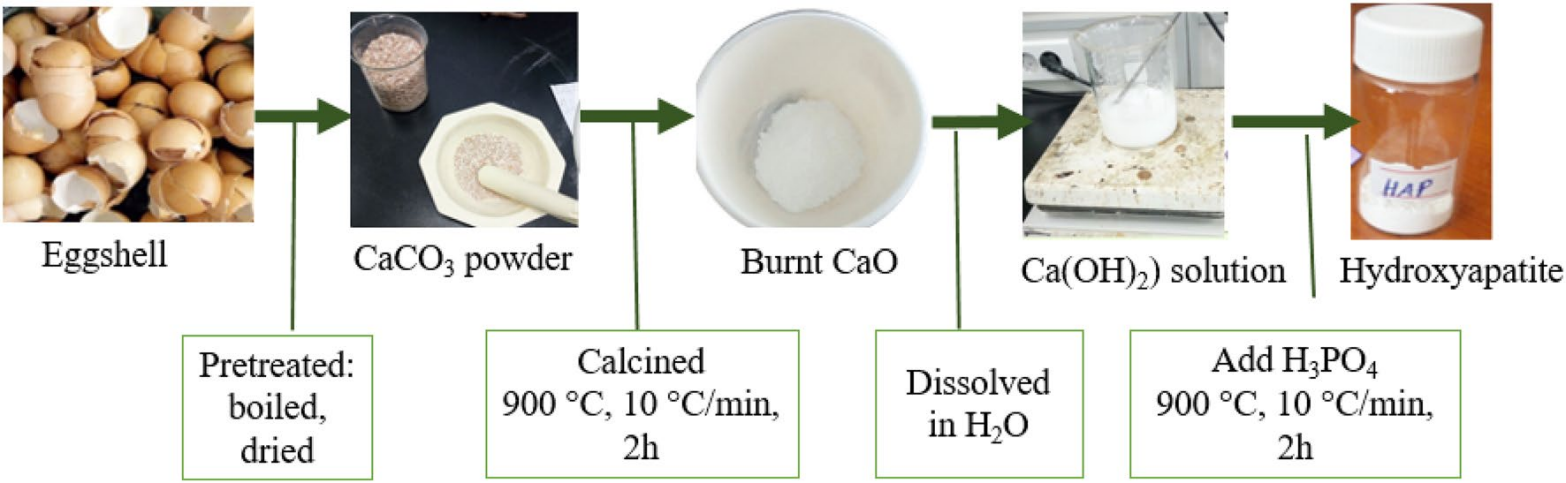
Synthesis steps of HAp from the eggshell.

HAp extracted from eggshell is environmentally friendly and cost-effective. HAp was extracted from eggshell by wet chemical precipitation method according to a procedure we reported elsewhere^27^. Figure 1 shows an overall extraction process of HAp from the eggshell.

### Synthesis of Bentonite/hydroxyapatite composite

In a particular method, 0.5 g HAp and 0.5 g bentonite were added in different beakers (250 mL) containing distilled water and stirred at room temperature for 10 h to produce a homogeneous suspension. HAp solution was then poured into the bentonite clay solution under continuous stirring. Then, 50 mL of glutaraldehyde was added dropwise to the mixture to form crosslinking between the hydroxyl and the aldehyde groups. Then, the (0.1N) HCl solution was added dropwise to hold the pH at about 3 and stirred for 12 h. The obtained product was washed with distilled water and ethanol until the pH of the solution became neutral and then dried at a temperature of 60 °C overnight.

### Characterization of the adsorbents

Shimadzu XRD- 7000 was used to analyze the phase purity of the adsorbents. Scanning electron microscopy (SEM, COXIEM-30, Shimadzu) was used for surface morphology characterization. The functional groups of the samples were characterized using Fourier-transform infrared spectroscopy (FT/IR-4000 series, JASCO). The textural properties of the adsorbent were measured by Brunauer–Emmett–Teller (BET) using a Quantachrome analyzer (Nova station C, version 11.0) based on the principle of adsorption/desorption of nitrogen at 77.3 K and 60/60 s (ads/des) equilibrium time.

### Optimization of removal of cadmium using RSM: ANOVA result

The experiments were carried out to optimize the selected input variables such as initial concertation of Cd (II), adsorbent dosage, solution pH, and contact time and further investigate their interactive effects on the adsorption efficiency of Cd (II) from aqueous solution onto bentonite/HAp composite. All statistical analyses for the optimization of experimental studies were performed using CCD under RSM.

The number of experiments required for a total of four parameters was determined from the following equation:

1 $$N={2}^{n}+2n+{n}_{c}$$

where N is the total number of experiments required, n is the number of factors, and n_c_ is the center point. Since a total of 30 experimental runs were conducted in this work, 2^4^ = 16 cube points, six replications at the center point, and eight axial points. Table 1 shows the level of chosen input variables and experimental ranges for Cd (II) removal efficiency (η).

**Table 1:** Levels of the adsorption process parameters considered.

Variables	Variable coding	Units	-alpha	Low	Center point	High	+ alpha
Initial Cd (II) conc	A	mg/L	25	50	75	100	125
Adsorbent dosage	B	g/L	0.25	0.75	1.25	1.75	2.25
pH	C	–	4.5	5	5.5	6	6.5
Contact time	D	min	30	40	50	60	70

The obtained experimental data were analyzed using Design expert 13.0 software, and the response variable (η) was correlated with four input factors by using the following quadratic polynomial equation:

2 $$\eta ={a}_{0}+\sum_{j=1}^{4}{a}_{j}{Y}_{j}+\sum_{j=1}^{4}{a}_{jj}{Y}_{J}^{2}+\sum_{I}\sum_{<j=2}^{4}{a}_{ji}{Y}_{i}{Y}_{j}+{e}_{i }$$

where η is the response (removal efficiency for Cd (II)); Y_i_ and Y_j_ refer to variables (i and j range from 1 to k); a_0_ refers to the coefficient of intercept; a_j_, a_jj_, and a_ij_ are known to be coefficients of interaction for the variables, respectively; and e_i_ is the error.

### Cadmium adsorption experiments

The adsorption experiment was carried out in batch mode. The synthetic solutions at different concentrations were prepared by diluting the standard Cd solution (1000 mg/L) obtained by dissolving CdCl_2_ in distilled water at room temperature. A sample of 20 mL volume adsorbate solution of specified initial concentrations (25–125 ppm) was shaken at 250 rpm at 25 °C using 1.25 g/L of the adsorbents (HAp/bentonite composite) in a 100 mL conical flask. Then, the samples were filtered using Whatman filter paper qualitative circles (125 nm $\varnothing \times 100$ circles) and analyzed the filtrates by an atomic absorption spectrometer (ZEEnit 700P, Atomic Flame Mode, Analytik Jena). All tests were carried out with repeatability, and their mean values were used in analyzing the data. The removal efficiency (η) was calculated using the following equation^13^.

3 $$\eta =\frac{({C}_{o}-{C}_{t})}{{C}_{o}}\times 100$$

The adsorption capacity *q*_e_ (mg/g) of the adsorbent was calculated according to the following equation.

4 $${q}_{e}=\frac{({C}_{o}-{C}_{t})V}{m}$$

where *C*_*o*_ (mg/L) is the initial concentration and *C*_*e*_ (mg/L) is the concentration of Cd (II) at equilibrium time. *V* (L) is the volume of the solution, and *m*(g) is the adsorbent mass.

### Adsorption isotherm analysis

In this study, the applicability of isotherm models (Freundlich and Langmuir) was tested to select the most fitting model that would best describe and predict the adsorption of Cd (II) onto HAp/bentonite.

The Freundlich isotherm was based on the assumptions of the monolayer adsorption process with heterogeneous surface energy: the adsorbed species interact with one another^28^. The well-known equation of this model can be given by the following equation^29^:

5 $${\mathrm{lnq}}_{\rm{e}}={\mathrm{lnK}}_{\rm{F}}+\frac{1}{\mathrm{n}}{\mathrm{lnC}}_{\rm{e}}$$

where *K*_*F*_ is the Freundlich constant which implies the affinity of the heterogeneous surface towards the adsorbet and 1/*n* verifies the favorability of the adsorption process.

The Langmuir isotherm model assumes that the adsorption occurs on homogeneous surfaces without interaction between the adsorbed Cd (II)^30^, and can be expressed according to the following Eq. (6) below.

6 $$\frac{{C}_{e}}{{q}_{e}}=\frac{1}{{q}_{max}b}+\frac{1}{{q}_{max}}{C}_{e}$$

where *q*_max_ (mg/g) is the monolayer adsorption capacity of the adsorbent, *b* (1/mg) is the Langmuir adsorption constant represents the affinity of the binding site.

Isotherms parameters for the two models were estimated from the slope and intercept of Eqs. (5) and (6) and listed in Table 6.

The favorability of Langmuir isotherm can be described by separation factor (R_L_) whether it is favorable (0 < *R*_*L*_ < 1), unfavorable (R_L_ > 1), irreversible (R_L_ = 0), or linear (R_L_ = 1) as described in the following Eq. 7:

7 $${R}_{L}=\frac{1}{1+{bC}_{o}}$$

where *Co* (mg/L) is the maximum initial Cd concentration and *b* (L/mg) is the Langmuir equilibrium constant^31^.

### Kinetic modeling

The pseudo-first-order model, pseudo-second-order model, intraparticle diffusion model, and double constant equation model were considered to investigate the reaction order and mechanism of the adsorption process. These models were calculated according to Eqs. (8–11), respectively^32,33^.

8 $$\mathrm{log}\left({q}_{e}-{q}_{t}\right)=\mathrm{log}{q}_{e}-\frac{{K}_{1}}{2.303}t$$

9 $$\frac{t}{{q}_{t}}=\frac{1}{{K}_{2}{q}_{e}^{2}}+\frac{1}{{q}_{e}}t$$

10 $${q}_{t}={K}_{i}{t}^{0.5}$$

11 $${lnq}_{t}=lnA+Blnt$$

where, *q*_e_ (mg/g) and *q*_t_ (mg/g) are the adsorption capacity at equilibrium and at time t (min), respectively, *K*_1_ (1/min) is the rate constants of pseudo-first-order; K_2_ (g/(mg·min)) is the rate constant of pseudo-second-order; k_i_ [mg/(g min^0.5^)] is the intraparticle diffusion rate constant; A and B are the double constant equation model constants. The kinetic constants and correlation coefficients for the models were calculated using the above equations and listed in Table 8 along with their R^2^ value.

## Results and discussions

### XRD analysis

**Figure 2:**
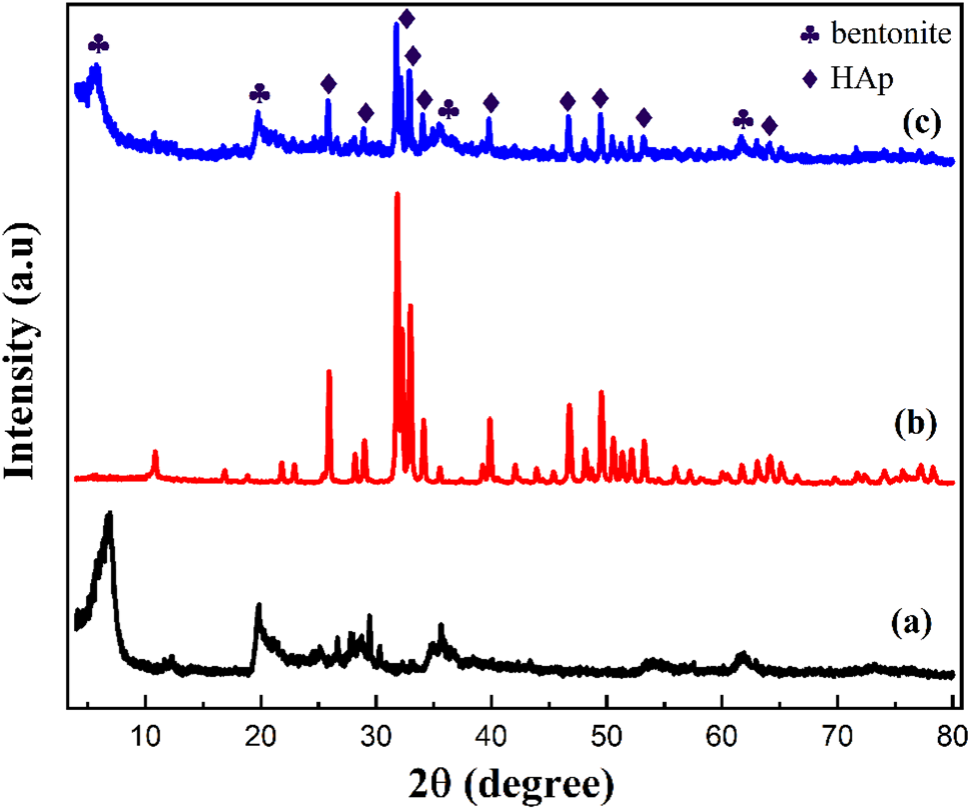
XRD pattern of (**a**) bentonite, (**b**) HAp, and (**c**) HAP/ bentonite composite.

Figure 2a–c shows XRD patterns of bentonite, HAp, and bentonite/HAp samples, respectively. In Fig. 2c, the sharp and major peaks related to HAp were observed at 2θ = 25.94,28.7 29.02,31.77, 32.19, 32.90, 34.05, 35.48, 39.20, 39.8, 42.03, 43.80, 45.30, 46.71, 48.10, 48.62, and 49.45 are related to (002), (102), (210) (211), (112), (300), (301), (212), (310), (311), (113), (203), (222), (312), (320), and (213) planes, respectively. The positions and d-values matched well with (the JCPDS pattern 09–0432)^34^. These peaks are also the same as those observed in Fig. 2b. However, the peaks at a 2θ = 6.6°, 20.1°, 35.7°, 54.1°, and 61.8°, which correspond to the (001), (100), (105), (210), (300) crystallographic planes, respectively (JCPDS No.12-0204)^35^, confirmed the presence of bentonite clay in the composite. Similar peaks can be observed for pristine bentonite particles as shown in Fig. 2a. Therefore, the formation of the HAp/bentonite composite was corroborated by XRD analysis. The crystallite size was estimated by Scherrer’s equation from the XRD pattern, D = $K\lambda /\beta \mathrm{cos}\theta$, where the constant k is 0.89, λ is the X-ray wavelength, β is the full-width half maximum and θ is the Bragg’s angle. The crystallite sizes of HAp/bentonite were estimated to be 42.03 nm.

### SEM analysis

**Figure 3:**
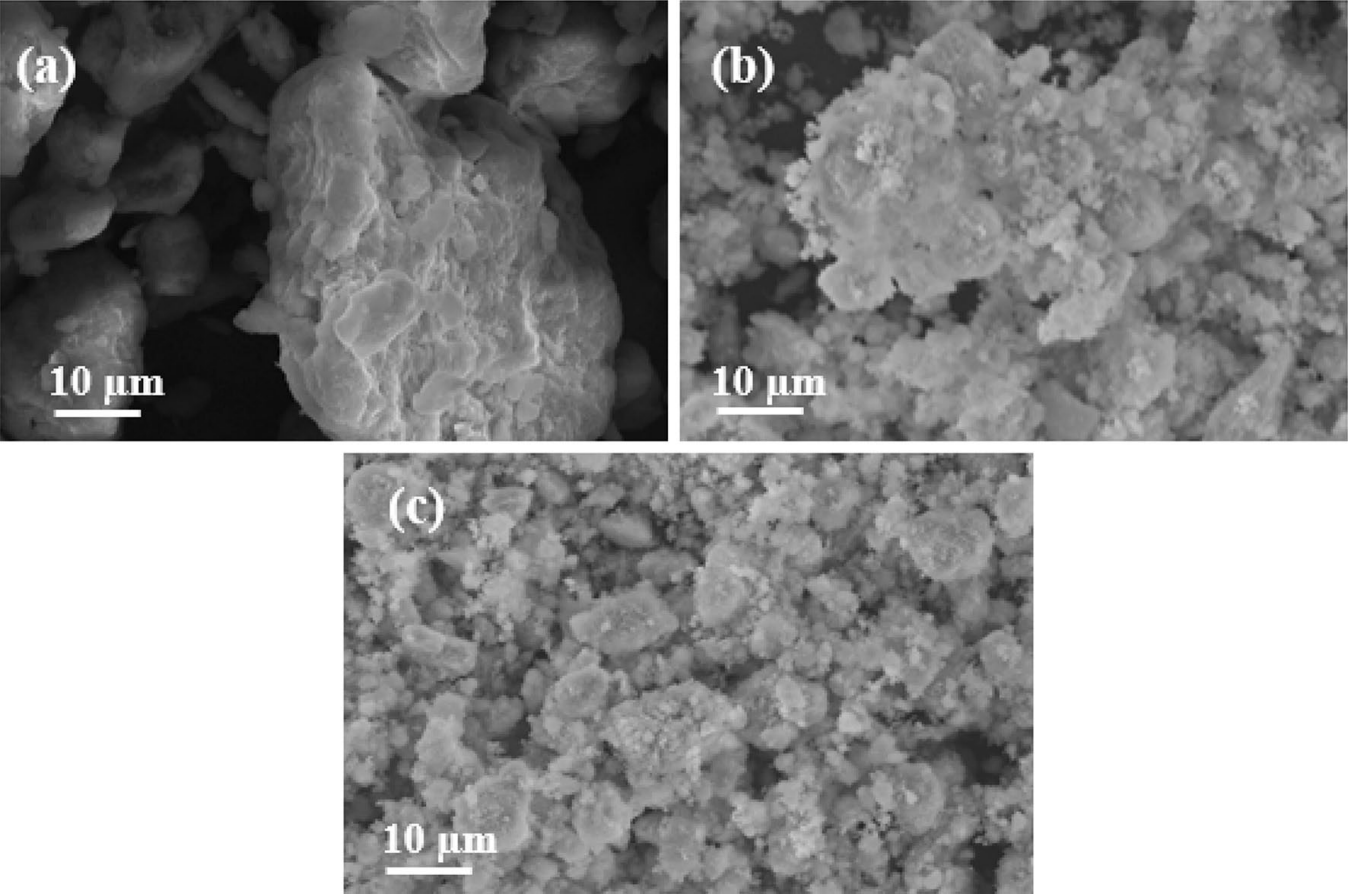
SEM image of (**a**) bentonite, (**b**) HAp**/**bentonite, and (**c**) HAp**/**bentonite composite after adsorption of Cd(II).

The surface morphology of bentonite, HAp/bentonite, and HAp/bentonite composite after adsorption of Cd(II) are shown in Fig. 3a–c, respectively. In Fig. 3a, the surface morphology of bentonite is a smooth surface with a fused and densely packed structure while the microstructure of HAp/bentonite composite revealed a rough and slightly porous surface. The SEM picture of HAp/bentonite also shows that the porous HAp aggregates are heterogeneously distributed over the surface of bentonite clay; hence, it provides more residence to the Cd(II) and has extra available bonding sites that facilitate the adsorption process. Figure 3c shows the morphology of the HAp/bentonite composite after Cd(II) adsorption. The morphology revealed an even greater degree of roughness on the surface due to the formation of thick assembly and adsorption of Cd(II).

### FTIR analysis

**Figure 4:**
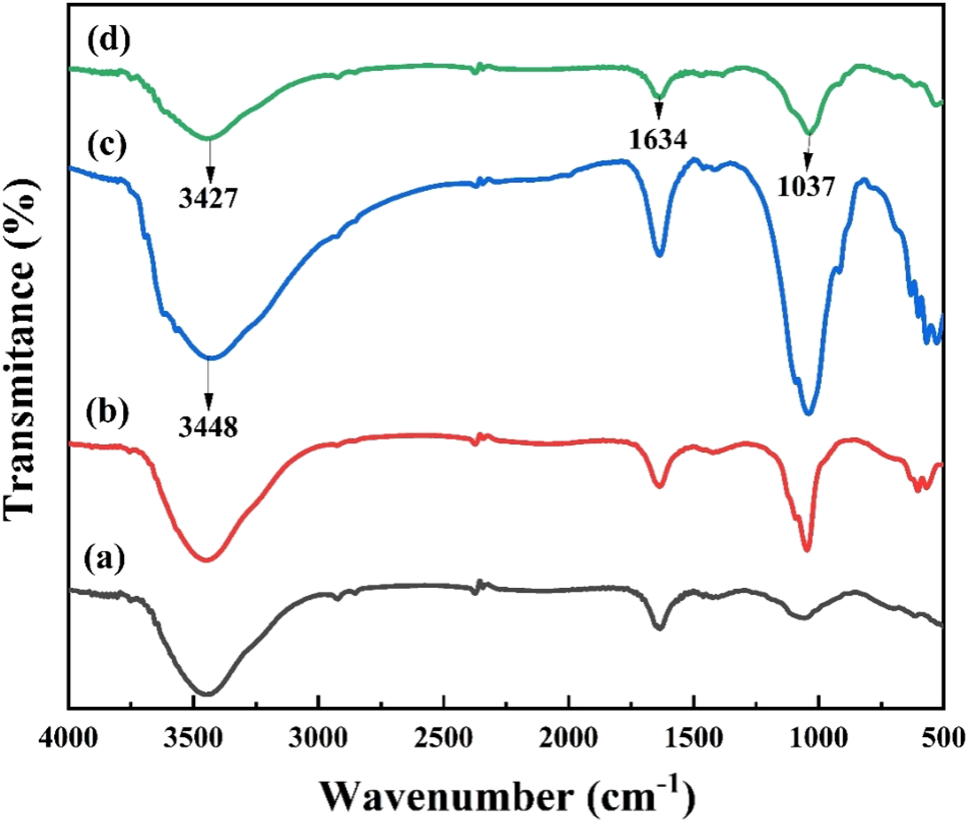
FTIR spectra of (**a**) bentonite, (**b**) HAp, (**c**) HAp/bentonite, and (**d**) HAp/bentonite after adsorption of Cd(II).

Figure 4a–d exhibits FTIR spectra of bentonite, HAp, HAp/bentonite, and HAp/bentonite composite after Cd(II) adsorption, respectively. Initially, the band around 1640 cm^−1^ and 3448 cm^−1^ were attributed to the hydroxyl group (–OH) stretching and bending, respectively in all samples. The number of water molecules present on the surface of the materials could be determined by their band intensity^27^. As shown in Fig. 4a the intensity of the band was relatively lower for bentonite, which implies it has fewer bonded H_2_O molecules. However, the peak of the band for HAp/bentonite composite was increased, which is credited to the presence of the large number of active hydroxyl groups on HAp/bentonite composite suggesting that the composite is favorable for the adsorption of Cd (II). The occurrence of PO4^3−^ was indicated by the appearance of a band at 1037 cm^−1^ confirming the successful loading of HAp on the bentonite surface^36^. A typical bentonite peak at 633 cm^−1^, displayed the typical vibrations of alumina and silica-oxygen tetrahedral groups. Furthermore, the characteristic peak at 522–527 cm^−1^ indicates the presence of Si–O–Si bonds in HAp/bentonite composite^37^. As shown in Fig. 4d the FTIR spectrum of the HAp/bentonite composite after Cd(II) adsorption was changed; the peaks at 3448 cm^−1^ and 1640 cm^−1^ attributed to the hydroxyl group (–OH) are relatively smaller than that of the sample before adsorption. This may be due to the complex formation between hydroxyl groups on the surface of HAp/bentonite and Cd(II) in solution^38^. The peak at 3448 cm^−1^ was also shifted to 3427 cm^−1^ after adsorption this shift may be caused by the presence of O–Cd(II) bonds. Moreover, the peak of P–O at 1037 cm^−1^ gets weakened, suggesting that phosphate participated in the adsorption process of Cd(II).

### BET analysis

The surface area was measured by absorption–desorption of nitrogen by the multipoint BET method. Concerning the textural properties, the composite presented a surface area of 257.27 m^2^/g and was characterized as a mesoporous material. Theoretically, the observed high surface area is good in adsorption as it provides more active sites on the surface of the adsorbent. Table 2 revealed the BET surface area, pore volume, and pore size of the adsorbent.

**Table 2:** Textural properties of prepared HAp/bentonite adsorbent.

Adsorbent	BET surface area (m^2^/g)	pore volume (cc/g)	pore diameter (nm)
HAp/bentonite	257.27	0.1621	7.92

### Central composite design (CCD) and analysis of variance (ANOVA)

CCD of the RSM approach was used to choose the effective model for the process and to optimize the adsorption process parameters^39–41^. The experiments were performed as per the experimental design set by RSM (Table 3).

**Table 3:** Batch mode adsorption process parameters used in RSM with the equivalent experimental and predicted values for the response.

Independent variables Cd removal efficiency (%, η)							
Std	Run	A	B	C	D	Efficiency	Predicted
19	1	75	0.25	5.5	50	74	73.65
15	2	50	1.75	6	60	94	95.39
6	3	100	0.75	6	40	78.5	79.18
18	4	125	1.25	5.5	50	77	76.35
16	5	100	1.75	6	60	92.3	92.47
3	6	50	1.75	5	40	94.5	95.89
4	7	100	1.75	5	40	90.3	90.07
30	8	75	1.25	5.5	50	98	97.25
27	9	75	1.25	5.5	50	96.5	97.25
14	10	100	0.75	6	60	81.5	81.37
17	11	25	1.25	5.5	50	87.9	88.06
29	12	75	1.25	5.5	50	97.5	97.25
26	13	75	1.25	5.5	50	97.5	97.25
25	14	75	1.25	5.5	50	96.5	97.25
2	15	100	0.75	5	40	74.6	74.47
1	16	50	0.75	5	40	84.2	83.27
13	17	50	0.75	6	60	87.8	87.27
7	18	50	1.75	6	40	97.3	95.95
28	19	75	1.25	5.5	50	97.5	97.25
23	20	75	1.25	5.5	30	91.9	91.88
9	21	50	0.75	5	60	84.8	85.70
21	22	75	1.25	4.5	50	88	87.93
20	23	75	2.25	5.5	50	97.5	97.36
8	24	100	1.75	6	40	92.3	92.65
5	25	50	0.75	6	40	84.7	85.45
24	26	75	1.25	5.5	70	94.6	94.13
11	27	50	1.75	5	60	97.4	95.95
22	28	75	1.25	6.5	50	92.5	92.08
12	29	100	1.75	5	60	90	90.50
10	30	100	0.75	5	60	76.7	77.28

The analysis of variance (ANOVA) was applied to check the statistical adequacy of the quadratic model (Table 4). The significance of each term in the quadratic model is determined by the model F and P- values^42^. In general, a higher F-value and lower P-value of a variable revealed the significance of the model terms^43^. According to the ANOVA results, the F-value for removing Cd(II) is 124.57 implies the model is statistically significant. Also, the P-value is less than 0.0500 indicating the model terms are significant. The coefficient of determination R^2^ was found to be 0.9915 which is close to one, implying that 99.15% of the variability in the response can be explained by the quadratic model, and therefore only 0.85% of the observed variability is insignificant by the model. Statistically, the difference between predicted R^2^ and adjusted R^2^ should be less than 0.2. In this case, the predicted R^2^ of 0.9556 is in reasonable agreement with the adjusted R^2^ of 0.9835; i.e., the difference is less than 0.2 and therefore is acceptable. Adequate precision measures the response values at the design space with the average prediction error, and a ratio greater than 4 is desirable. In this study, the Adeq precision is 34.1168 indicating an adequate signal, that can be used to navigate the design space. Besides the aforementioned results, a quadratic model is chosen to represent the interaction of the process variables as given in Eq. (10)^44^.

12 $$\eta =97.25-2.93A+5.93B+1.04C+0.5625D+0.7438AB+0.6313AC-0.5312BC-0.5937BD-{3.76A}^{2}-{2.94B}^{2}-{1.81C}^{2}-{1.06D}^{2}$$

where η (%) is the removal efficiency of Cd(II), A is the initial concentration, B is the dosage of the adsorbent, C is the pH and D is the contact time.

**Table 4:** ANOVA results for Cd removal using a quadratic model from CCD.

Source	Sum of squares	df	Mean square	F-value	p-value	
Model	1685.60	14	120.40	124.57	< 0.0001	Significant
A	205.92	1	205.92	213.06	< 0.0001	
B	843.72	1	843.72	872.96	< 0.0001	
C	25.83	1	25.83	26.73	0.0001	
D	7.59	1	7.59	7.86	0.0134	
AB	8.85	1	8.85	9.16	0.0085	
AC	6.38	1	6.38	6.60	0.0214	
AD	0.1406	1	0.1406	0.1455	0.7082	
BC	4.52	1	4.52	4.67	0.0472	
BD	5.64	1	5.64	5.84	0.0289	
CD	0.3906	1	0.3906	0.4042	0.5345	
A^2^	388.08	1	388.08	401.53	< 0.0001	
B^2^	236.51	1	236.51	244.71	< 0.0001	
C^2^	90.00	1	90.00	93.12	< 0.0001	
D^2^	30.90	1	30.90	31.97	< 0.0001	
Residual	14.50	15	0.9665			
Lack of fit	12.62	10	1.26	3.37	0.0963	Not significant
Pure error	1.88	5	0.3750			
Cor total	1700.10	29				

**Figure 5:**
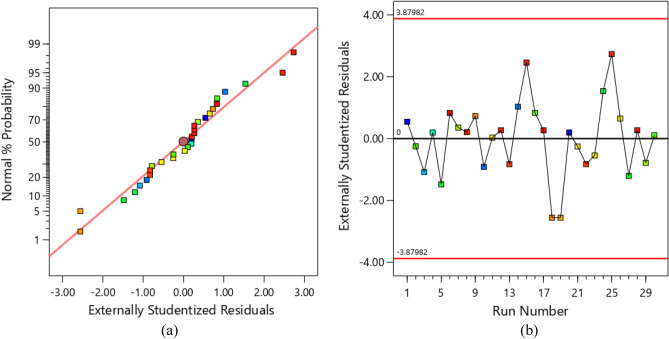
The normal plot of residuals (**a**), the plot of residuals versus run number for removal of Cd(II) (**b**).

To evaluate the selected model, an analysis of the residuals was performed. Residuals can be defined as the difference between the experimental and predicted value by the model^45^. Figure 5a shows the normal probability versus externally studentized residuals for the removal efficiency of Cd(II). As can be seen, the residuals follow a straight line with no deviation. The plot shows a normal distribution of errors, confirming the significance of the selected models. In addition, Fig. 5b indicates a random scatter of residuals compared to the expected values. The plot revealed that the points distributed along a straight line, the significance of the model.

#### Effect of selected variables on the removal of Cd (II)

**Figure 6:**
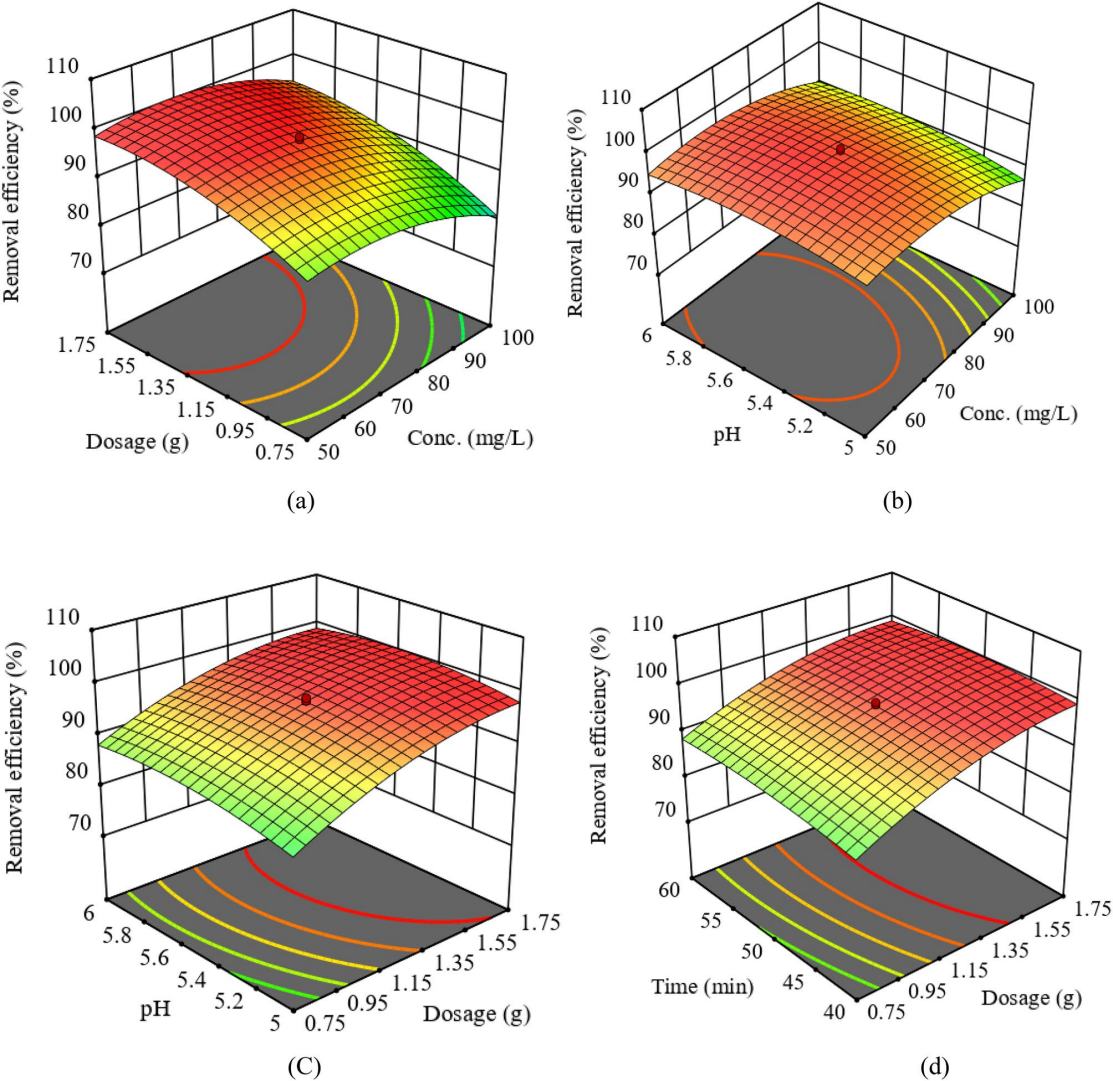
Interactive effect of parameters on the removal efficiency of Cd (II) (**a**) initial Cd concentration versus adsorbent dosage, (**b**) initial Cd concentration versus pH, (**c**) adsorbent dosage versus solution pH, and (**d**) adsorbent dosage versus contact time.

The interaction of selected process parameters towards the adsorption of Cd(II) was investigated and shown in Fig. 6a–d. The interactive effect of the initial Cd(II) concentration and the adsorbent dose on the removal efficiency of the adsorbent was shown in Fig. 6a. From the 3D surface plot, it is clearly stated that the adsorption capacity decreased with increasing Cd(II) concentration. An increase in Cd(II) concentration from 50 to 100 mg/L with a 1.58 g/L dose resulted in a decrease in adsorptive removal from 99.3 to 80%. This could be attributed to the fact that the surface of the HAp/bentonite composite becomes saturated in the presence of a higher concentration of Cd(II), eventually, the Cd(II) ion tends to form an aggregate on the outer surface of the composite and deceased the surface area. Similar results have been reported by Praipipat et al (2023), and the study showed that the removal efficiency was decreased with increasing concentration^46^.

On the other hand, the sorption capacity increased with increasing the dosage of the composite^47^. The probable reason is that when the adsorbent dose increases, the active sites of HAp/bentonite increase until all of the Cd(II) ions are adsorbed on the surface^48^. These results are in line with Muhammad Shafiq et al. (2022), who studied the removal of Cd(II) from contaminated water using functionalized bentonite clay composite with NiAl-Layered Double Hydroxide. According to the obtained results, an increase in the uptake capacity of Cd (II) was observed by increasing the dosage of the adsorbents^49^. Another result has been reported by Zongqiang Zhu et al. (2022), who investigated the removal of Cd(II) using Strontium-doped hydroxyapatite as an adsorbent. The effect of adsorbent dosage, temperature, and an initial solution was studied. The result revealed that with increasing adsorbent dosage, the removal efficiency increased^50^.

Figure 6b depicts the 3D plot of the initial Cd(II) concentration and the solution pH i.e., the pH has a positive effect on the removal efficiency. At a given pH of 5.8, an increase in Cd(II) concentration from 50 to 100 mg/L resulted in a decrease in the removal efficiency from 99.3 to 92%.

The 3D surface plot presented in Fig. 6c stated the effect of the adsorbent dose and the solution pH on Cd(II) removal. It was revealed that the removal efficiency increases with increasing the adsorbent dosage and the optimum dose was considered to be 1.58 g/L. From the 3D plot, it was also observed that the adsorptive removal efficiency increases from 90 to 99.3% upon increasing the pH. As can be seen from Fig. 10, the pHpzc of HAp/bentonite composite is 5.9. i.e., at pH < 5.9, the surface of the composite is positively charged and at pH > 5.9, the surface of the composite is negatively charged. Therefore, the removal efficiency decreases at pH < 5.9. This is because, at lower pH, Cd(II) and H^+^ ions compete to occupy the binding sites on the adsorbent surface, which leads to decreased adsorption capacity^51,52^. On the contrary, in the basic medium, the surface HAp/bentonite composite becomes negatively charged, thereby decreasing the repulsive force. Although, with a further increase in pH, the Cd(II) tends to precipitate as Cd(OH)_2_. These results found support from Abdelbaky Hossam Elgarhy et al. (2022), who demonstrated that the removal percentage efficiency increased with increasing pH value. The findings further showed that the cation exchange capacity is strongly controlled by the pH of the solution^53^.

Figure 6d presents the effect of varying the adsorbent dosage and contact time. At low contact time, the percentage removal of Cd(II) was fast. This is due to the presence of a pristine active site in the initial stage of adsorption followed by a slight increase in removal efficiency. However. the addition of time no longer increases the percentage removal after the contact time of 50 min, this is due to the active sites on the adsorbent becoming saturated by the Cd(II), and in time the Cd(II) detached from the surface of the adsorbent back into the solution^54^. Hao Xu et al. (2020) obtained a similar result in their study to remove Cd(II) and Pb(II) using humic acid-iron-pillared bentonite. The result revealed that the adsorption capacity increased with the increase in contact time, reached a plateau region, and then slightly declined^55^.

#### Optimization of removal of Cd (II) using RSM

The optimum values of the process parameters for the maximum Cd removal efficiency were determined as shown in Table 5 below. The experimental removal efficiency of HAp/bentonite at that optimal condition was proximate to the predicted value. This proves that the adopted method to optimize process parameters for maximum adsorption of Cd(II) onto HAp/bentonite composite is successful.

**Table 5:** Optimal conditions of the selected factors for Cd adsorption via HAp/bentonite.

Optimized process parameters				Removal efficiency (η, %)		
Initial Concentration	Dosage	pH	Time	Predicted	Actual	Desirability
61.58	1.58	5.88	49.63	99.3	97.85	0.987

#### Adsorption equilibrium isotherm

**Figure 7:**
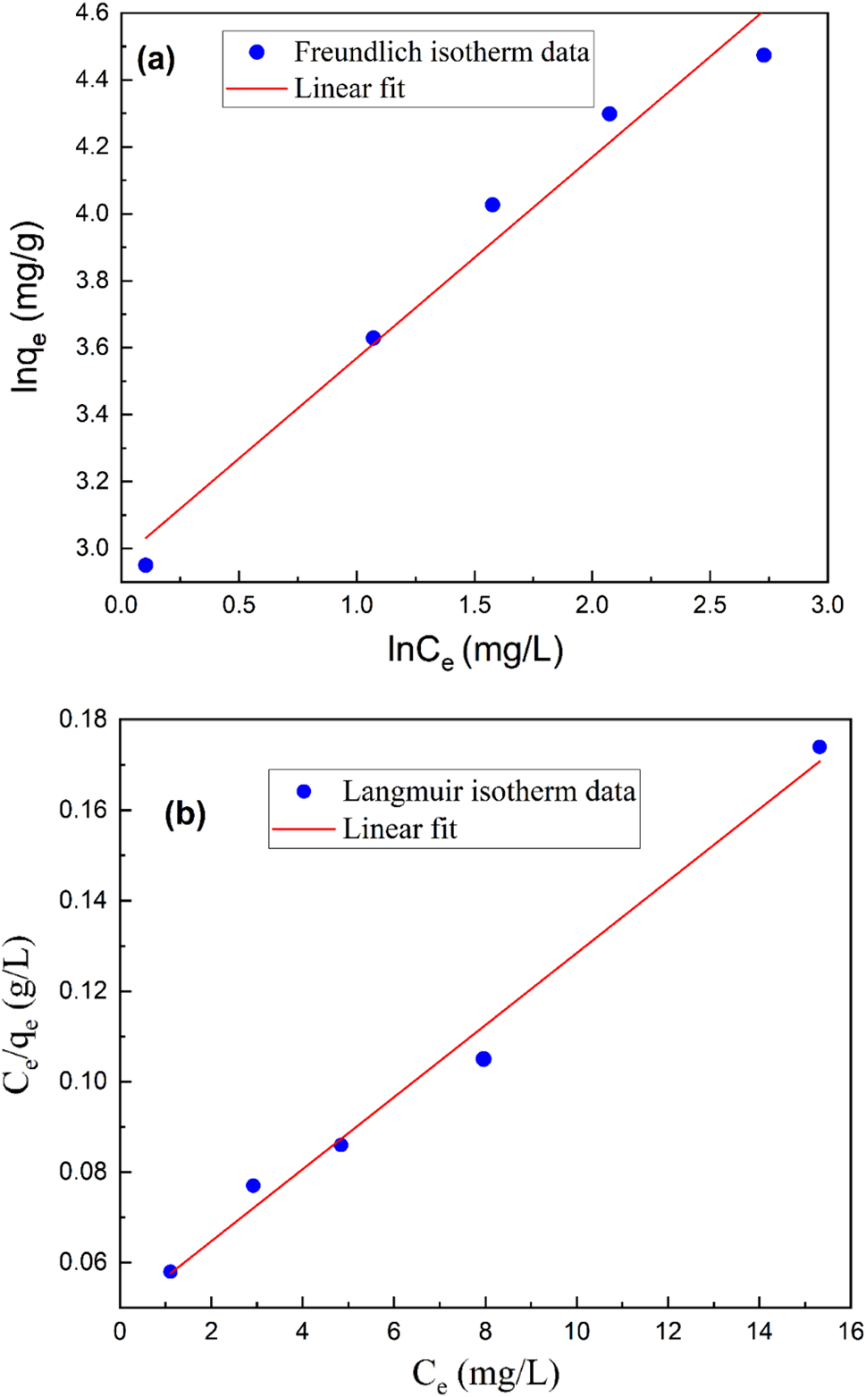
Freundlich (**a**) and Langmuir isotherms (**b**) for adsorption of Cd(II) onto HAp/bentonite composite.

Figure 7 depicts a comparison of the equilibrium data with the Langmuir and Freundlich isotherms to understand the sorption behavior^56^. Table 6 suggests a better (R^2^ = 0.9889) value for adsorption data of HAp/bentonite composite for the Langmuir adsorption isotherm model than the Freundlich model, thus, proving the homogeneous adsorbents. The separation factor R_L_ of the Langmuir isotherm model suggests the favorability of the adsorption system. The value of R_L_ should be comprised between 0 to 1 for favorable adsorption^57^. In this study, the value of *R*_*L*_ was 0.05; confirming the applicability of the Langmuir isotherm. The maximum sorption capacity was found to be 125.47 mg/g and that was significantly higher compared to other adsorbents for Cd(II) removal from aqueous solutions as shown in Table 7.

**Table 6:** Parameters of Freundlich and Langmuir isotherms for adsorption of Cd(II) onto HAp/bentonite composite.

Isotherm models	Parameters	Values	*R*^*2*^
Freundlich	*n*	1.67	0.9700
	*K*_*F*_ (L/g)	19.47	
Langmuir	*q*_*max*_ (mg/g)	125.47	0.9889
	*b* (1/mg)	0.16	
	*R*_*L*_	0.05

**Table 7:** Comparison of maximum adsorption capacities of various adsorbents towards Cd(II).

Adsorbent	Maximum adsorption capacity (mg/g)	References
Ball clay	27.17	^58^
Bentonite–Fe_3_O_4_–MnO_2_	35.35	^59^
Composite of eggshell/starch/Fe_3_O_4_	48.57	^60^
Thiol-modified bentonite (SH-Bent)	49.3	^61^
Nano-hydroxyapatite-modified biochar (HBC)	55.95	^62^
Amino-functionalized bentonite/CoFe_2_O_4_@MnO_2_	115.79	^17^
Hydroxyapatite-encapsulated zinc ferrite (HAp/ZnFe_2_O_4_)	120.33	^63^
HAp/bentonite composite	125.47	Present study

#### Adsorption kinetics

**Figure 8:**
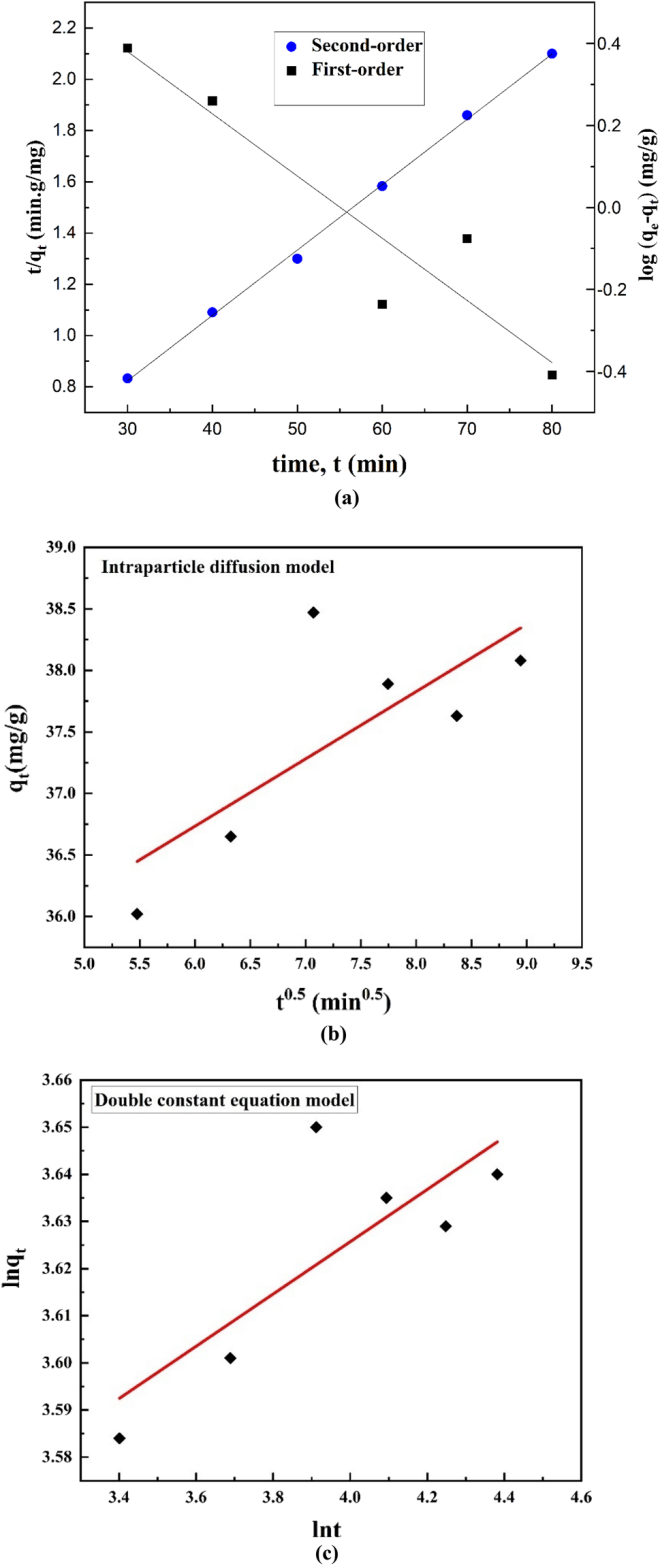
Linear fitting curves of adsorption kinetics. (**a**) Pseudo-first-order and pseudo-second-order kinetics models, (**b**) intraparticle diffusion model, and (**c**) double constant equation model for adsorption of Cd(II) onto HAp/bentonite composite.

The adsorption mechanism and rate were determined by the desired kinetic model, which in turn depends on the value of the correlation coefficient (R^2^) for the linear mode of different kinetic equations. The kinetic parameters and their associated R^2^ value are presented in Table 8, where R^2^ was used to compare the fitting of the kinetic curves. Figure 8a shows a plot of log(q_e_ − q_t_) versus t, and (t/q_t_) versus t according to the pseudo-first-order and pseudo-second-order models, respectively. The low R^2^ value of the pseudo-first-order model in Table 8 shows that the adsorption of the Cd(II) onto HAp/bentonite did not follow this model. However, the R^2^ value of the pseudo-second-order model was found to be very close to 1.0. Furthermore, the estimated q_e_ value for the adsorption of Cd(II) found from the pseudo-second-order model is closer to the experimental q_e_ value, which suggests that the pseudo-second-order model fits the adsorption process. In Fig. 8b, the fit of the intraparticle diffusion model was determined by the plot of q_t_ versus t^0.5^. The R^2^ value was 0.5760 implying that the adsorption did not follow this model. In Fig. 8c, lnq_t_ was plotted versus lnt according to the double constant equation model. R^2^ was also 0.6402, indicating that this model did not fit with the adsorption process. Therefore, it can be concluded that the adsorption of Cd(II) onto HAp/bentonite followed pseudo-second-order i.e., the rate involves forces for sharing or exchanging of electrons between Cd(II) and HAp/bentonite composite (chemisorption)^64^.

**Table 8:** R^2^ and constant values for different adsorption kinetics models of Cd(II) adsorption onto HAp/bentonite composite.

Model	Parameters	Values	*R*^2^
Pseudo-first-order	*q*_*e,exper.*_ (mg/g)	38.47	0.8866
	*q*_*e,calc*_. (mg/g)	6.844	
	*K*_1_ (min^−1^)	0.0350	
Pseudo-second-order	*q*_*e,calc*_. (mg/g)	39.21	0.9985
	*K*_*2*_(min^−1^)	0.0111	
Intraparticle diffusion	*K*_*i*_	0.5471	0.5760
Double constant equation model	*A*	30.081	0.6402
	*B*	0.0554

#### Adsorption thermodynamics

To understand the nature of Cd(II) adsorption on HAp/bentonite, the thermodynamic parameters such ash as free energy change (ΔG), enthalpy changes (ΔH), and entropy change (ΔS) were investigated according to Van’t Hof equation^65^.

13 $$\Delta G=-RTln{K}_{f }$$

14 $${K}_{f}=\frac{{q}_{e}}{{C}_{e}}$$

15 $$ln{K}_{f}=-\frac{\Delta H}{RT}+\frac{\Delta S}{R}$$

where K_f_ (L/g) is called the distribution constant, q_e_ (mg/g) is the equilibrium adsorption capacity, C_e_ (mg/L) is the equilibrium concentration of the solution, T(K) is the absolute temperature, and R is the universal gas constant (8.314 J/mol K).

**Figure 9:**
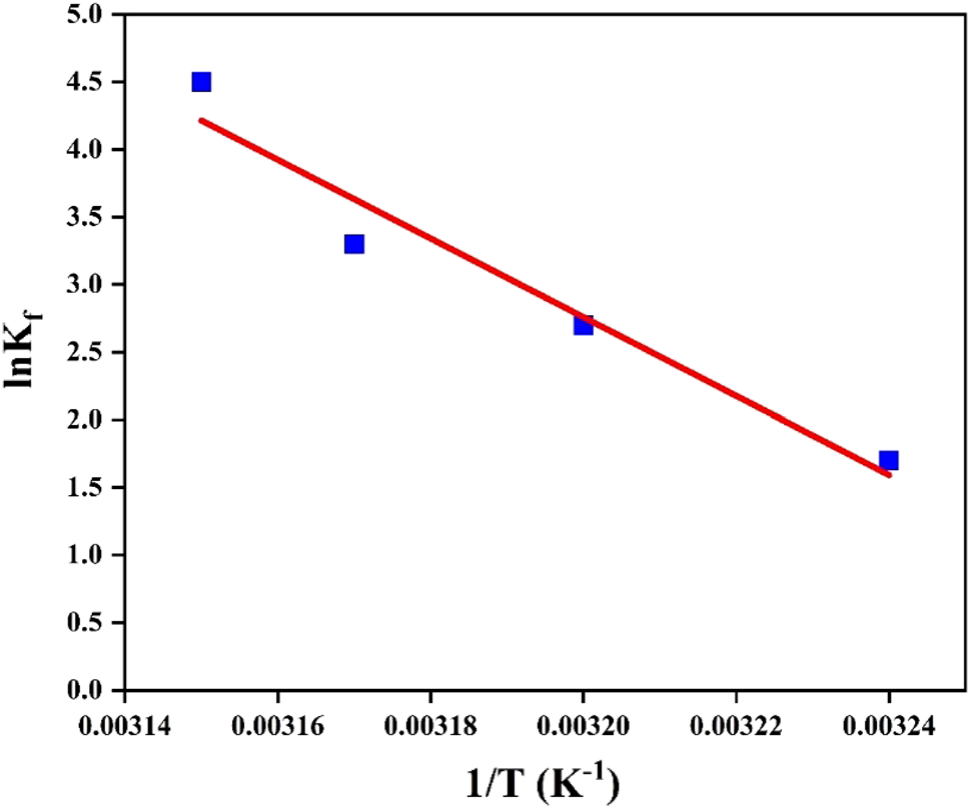
Adsorption thermodynamics of Cd(II) onto HAp/bentonite composite.

The Gibbs free energy (ΔG) of the adsorption reaction was calculated according to Eq. (11). Whereas the enthalpy change (ΔH) and the entropy change (ΔS) of adsorption were calculated from Van’t Hoff plot of lnK vs 1/T (Fig. 9). Thermodynamics results are shown in Table 9, where the positive value of ∆H indicates the adsorption process is endothermic due to the increase in adsorption upon a successive increase in the temperature. Negative values of ΔG indicate that the nature of Cd(II) adsorption is spontaneous and thermodynamically favorable. Moreover, the change in Gibbs free energy was inversely proportional to the temperature, and an increase in temperature enhanced the adsorption. ΔS value is negative, indicating that there is a higher disorder at the solid–the liquid interface during adsorption, which may be related to the ion exchange process^66^.

**Table 9:** Thermodynamic parameters of the adsorption of Cd(II) onto HAp/bentonite.

Adsorbent	*∆H* (kJ/mol)	*∆S* (Jmol/K)	*∆G* (kJ/mol)			
			25 °C	35 °C	45 °C	55 °C
HAp/bentonite	7148	254.10	−4.24	−6.78	−9.32	−11.86

#### The point of zero charge of HAp/bentonite composite

**Figure 10:**
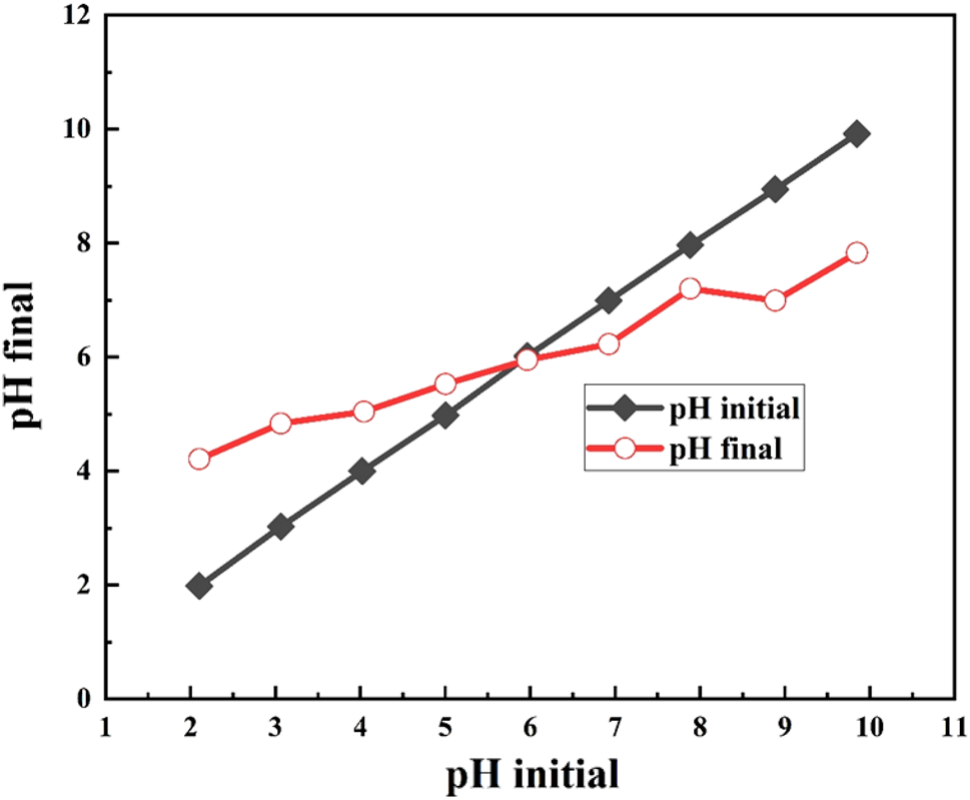
The pHpzc of HAp/bentonite.

The point of zero charge is the pH at which the total surface charge of the adsorbent is neutral^67^. In this study, the pH of the 0.01 M NaCl was adjusted to a value between 2 and 10 using 0.1 M HCl or 0.1 M NaOH. An adsorbent (0.5 g) was added to 50 mL of the pH-adjusted solution and stirred for 3 h on a shaker. Then, the pH of each solution was measured, and the diagram of the initial pH versus the final pH was plotted (Fig. 10). The pH where the two curves intersect is called pHpzc, which is equal to 5.9 for HAp/bentonite.

#### Proposed mechanism for the adsorption process

**Figure 11:**
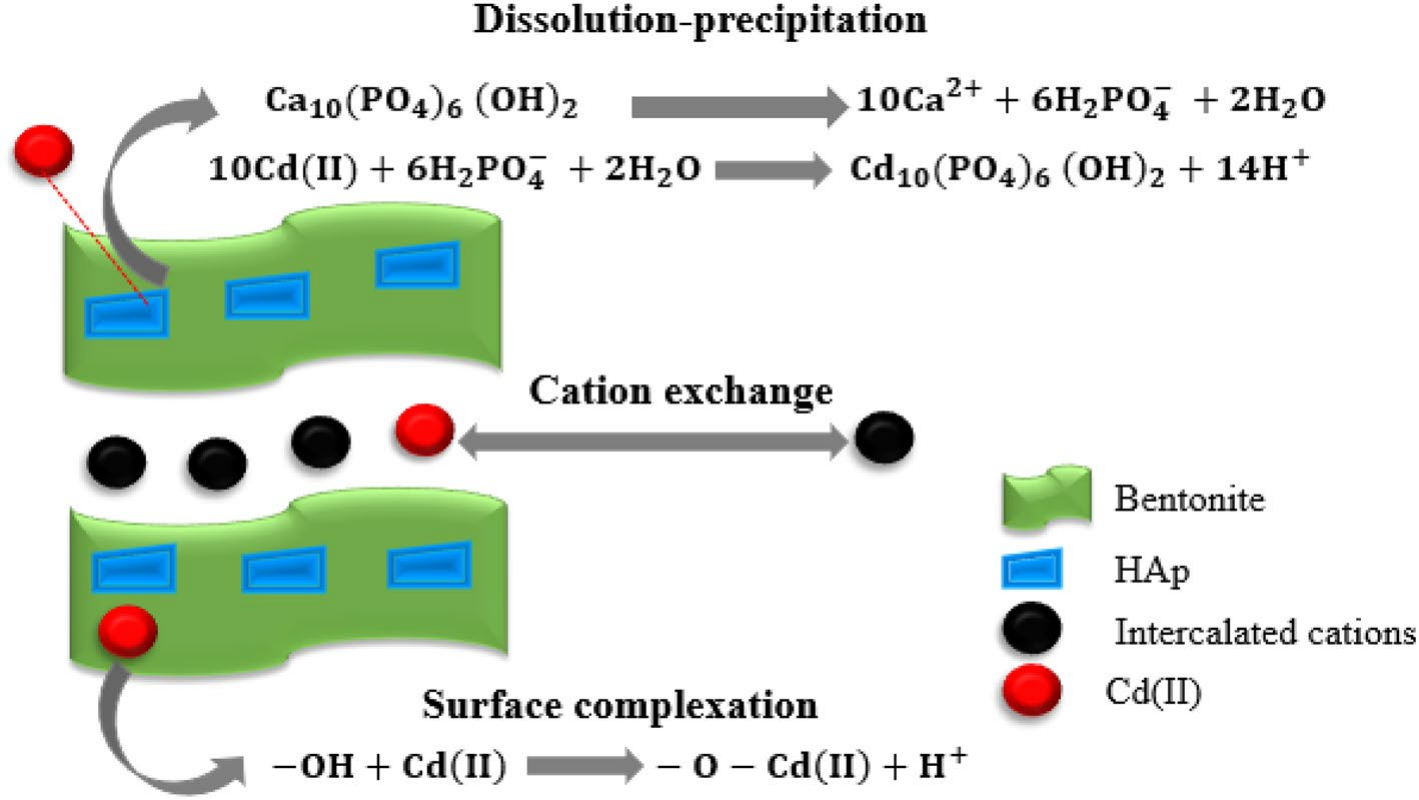
The possible mechanism of Cd(II) adsorption on HAp/bentonite composite.

As depicted in Fig. 4d, the FTIR peaks at 3440 cm^−1^ and 1640 cm^−1^ were reduced after Cd(II) on the composite, which implies surface complex formation between hydroxyl groups and Cd(II). Moreover, the adsorption peak of P–O at 1038 cm^−1^ was changed, which proves that the role of the phosphate group of HA_P_ was also significant. The adsorption of Cd(II) onto the HAp/bentonite surface relies on certain critical factors such as surface area, porosity, hydrogen bonding, electrostatic interactions between oppositely charged adsorbent surfaces and the adsorbate, etc^26^. Furthermore, the solution pH also played a significant role by enhancing the deprotonation of binding sites and hence boosting the adsorption of Cd(II). The functional surface possessing phosphate groups exhibits a high affinity towards Cd(II) by electrostatic interactions in addition to the negatively charged bentonite layers^68^. Generally, The possible adsorption mechanism of Cd(II) onto the HAp/bentonite was schematically explained as shown in Fig. 11.

## Conclusion

The preparation and testing of a HAp/bentonite composite for the removal of Cd(II) from an aqueous solution were both successful. A facile synthesis route is reported in this study, which considers cost-effective material resources and environmental control. XRD, SEM, FTIR, and BET techniques were used to characterize the composite. The CCD-based RSM was used to optimize important process parameters such as initial Cd(II) concentration, adsorbent dosage, PH, and contact time. Based on the analysis of variance (ANOVA) result, the quadratic model was confirmed to describe the removal efficiency with a high correlation coefficient (R^2^ = 0.9987). At optimal conditions, it was statistically optimized that 61.58 mg/L initial MO concentration, 1.58 g adsorbent dosage, 5.88 solution pH, and 49.63 min. contact time can achieve the maximum removal efficiency of 99.3%. Adsorption kinetics followed a pseudo-second-order kinetic model, indicating the chemisorption of Cd(II) on the surface of the composite. Equilibrium data were also best fitted with the Langmuir isotherm model, indicating that the surface active sites have an equal affinity towards Cd(II) ions. The maximum monolayer adsorption capacity was 125.47 mg/g, and the separation coefficient R_L_ was in the range of 0 to 1, indicating that HAp/bentonite was favorable for Cd (II) adsorption. Furthermore, thermodynamic studies illustrated that the adsorption of Cd(II) was a spontaneous and endothermic process. Meanwhile, the adsorption of Cd(II) onto HAp/bentonite is dependent on certain critical factors such as cation exchange, dissolution–precipitation, and surface complexation. In summary, the experimental results of this study showed that HAp/bentonite, which is safe, low-cost, environmentally friendly, and available material could be an effective adsorbent with optimized process parameters by CCD to remove Cd(II) from contaminated water.

## Data Availability

The datasets generated during and/or analyzed during the current study are available from the corresponding author on reasonable request.
